# Modeling Strategies for Neurodegenerative Diseases: In Vitro, In Vivo and In Silico Frameworks

**DOI:** 10.3390/biomedicines14102227

**Published:** 2026-10-01

**Authors:** Francesca Martorella, Camilla Cigolini, Giorgia Pupo, Tiziana Alberio, Marta Lualdi

**Affiliations:** 1 Department of Science and High Technology, University of Insubria, 21052 Busto Arsizio, Italy; fmartorella@uninsubria.it (F.M.); ccigolini@studenti.uninsubria.it (C.C.); gpupo@studenti.uninsubria.it (G.P.); tiziana.alberio@uninsubria.it (T.A.); 2 Department of Medicine and Technological Innovation, University of Insubria, 21100 Varese, Italy

**Keywords:** neurodegeneration, disease modeling, in vitro models, in vivo models, in silico models, personalized medicine

## Abstract

Neurodegenerative diseases (NDs) comprise a heterogeneous group of disorders characterized by complex etiologies, multifactorial pathogenic mechanisms, and remarkable clinical and molecular variability. Their increasing prevalence poses an important challenge to healthcare systems, society and the global economy. Despite decades of research, effective disease-modifying therapies remain elusive, highlighting the urgent need for reliable disease models capable of unraveling pathogenic mechanisms and supporting the development of personalized therapeutic strategies. Modeling NDs remains inherently challenging, since disease onset and progression arise from the interplay among genetic factors, environmental exposures, aging, and individual pathological conditions. Consequently, similar insults may lead to distinct molecular trajectories and clinical outcomes in different subjects. Reproducing this heterogeneity, together with the prolonged temporal evolution that characterizes NDs over decades in humans, represents a major limitation of current experimental models. Here, we provide a comprehensive overview of the modeling strategies currently available for the study of NDs, spanning conventional two-dimensional cellular systems, advanced patient-derived three-dimensional organoids, complementary in vivo animal models, and rapidly evolving in silico approaches. For each model category, we discuss its historical development, current applications, future perspectives, and, critically, principal strengths and limitations in reproducing disease biology and supporting translational research. We emphasize that the growing synergy between in vitro, in vivo, and in silico methodologies, empowered by multimodal data integration and artificial intelligence, is expected to accelerate biomarker discovery, improve disease modeling, reduce experimental burden, and ultimately foster the development of personalized disease-modifying therapies for NDs.

## 1. Introduction

Neurodegenerative diseases (NDs) are a heterogeneous group of complex multifactorial disorders affecting millions of people worldwide [1]. Disease prevalence varies considerably across disease categories, ranging from highly prevalent conditions, such as Alzheimer’s disease (AD) and other dementias, affecting approximately 57 million people worldwide, and Parkinson’s disease (PD), with nearly 12 million prevalent cases, to rarer disorders such as amyotrophic lateral sclerosis (ALS) and hereditary cerebellar ataxias, whose prevalence is generally in the order of a few cases per 100,000 individuals. According to the World Health Organization (WHO), the number of people living with dementia worldwide will be around 139 million by 2050 [2]. Beyond their devastating impact on patients and caregivers, NDs impose a substantial socioeconomic burden, with global costs estimated at over USD 1.3 trillion and expected to increase dramatically in the coming decades [2]. In this context, the development of reliable biomarkers for early diagnosis and the identification of effective disease-modifying therapies are urgent priorities.

NDs arise from complex interactions among genetic, environmental, and aging-related factors that ultimately converge on progressive neuronal loss, leading to impaired nervous system physiology and, consequently, dysfunction of the entire organism. NDs can arise either sporadically or as familial forms, with the latter often linked to specific pathogenic variants that accelerate disease onset or severity and shape the pathological phenotype. Familial forms often highlight pathogenic genes or molecular pathways that are also involved in sporadic cases, thereby providing valuable disease models. Clinically, NDs can be broadly classified into major categories: (i) dementias, including AD, frontotemporal dementia (FTD), and dementia with Lewy bodies; (ii) parkinsonian disorders and synucleinopathies, such as PD, multiple system atrophy, and progressive supranuclear palsy; (iii) motor neuron diseases, such as ALS and primary lateral sclerosis; (iv) cerebellar/spinocerebellar ataxias; and (v) hyperkinetic movement disorders, including Huntington’s disease (HD).

Despite clinical heterogeneity, major pathological hallmarks of NDs are synaptic and neuronal network dysfunction, neurotransmitter dyshomeostasis, aberrant proteostasis with the formation and spread of pathological protein aggregates, mitochondrial dysregulation, and neuroinflammation [3]. These overlapping disease mechanisms represent a major challenge for both patient stratification and early diagnosis, as disease-specific biomarkers are often difficult to identify. Another shared feature of NDs is that clinical symptoms frequently develop gradually over years or decades before becoming manifest [4], and by the time symptoms emerge, substantial neuronal and synaptic loss has already occurred, posing a major challenge to effective therapeutic interventions. Thus, treatments have historically relied largely on symptomatic and supportive approaches. Now, the therapeutic landscape is rapidly evolving, with the emergence of disease-modifying strategies aimed at targeting specific pathogenic mechanisms and potentially slowing disease progression. In this frame, personalized medicine approaches would ideally provide the optimal solution, tailoring treatments to an individual’s most prominent ND hallmarks [5]. However, identifying specific and robust biomarkers to precisely stratify and characterize individual patients is still far from being clinically feasible and applicable [6].

Adding further complexity, although neurons are the primary cell type affected and play a central role in disease onset and progression, it is now widely recognized that NDs should no longer be studied as neuron-centered diseases. Rather, they should be considered multi-level processes driven by the interplay between intrinsic neuronal vulnerability and systemic modulators (e.g., gut-microbiota-derived metabolites, systemic metabolic dysfunction, immune system, inflammatory mediators) [7].

In this context, one of the main problems in ND research is the difficulty of generating models that closely mimic the complexity of the disease [8]. In particular, the availability of preclinical ND models that capture disease heterogeneity, core molecular/cellular processes, and biochemical complexity presents a major challenge. Moreover, recapitulating the protracted temporal dynamics of NDs, which unfold over decades in humans, represents a critical limitation that proves difficult to overcome using both cellular and animal models [9].

In this narrative review, we provide a comprehensive overview of the major modeling approaches currently used to study NDs, encompassing established cellular in vitro models, advanced three-dimensional (3D) patient-derived organoid systems, complementary in vivo approaches (i.e., animal models), and emerging in silico platforms. We searched publicly available scientific literature repositories, focusing on review articles published over the last 10 years and original research articles published primarily within the last 5 years. Studies were selected based on their relevance to the development and/or application of the described modeling approaches to NDs, including examples of both established and emerging strategies. The predominant focus on AD and PD throughout this review reflects the substantially larger body of literature available for these disorders compared with other, less extensively investigated NDs. Nevertheless, whenever sufficient evidence was available, we also included representative modeling studies addressing less extensively studied NDs.

Rather than focusing on individual modeling strategies, we aim here to provide an integrated perspective on these different approaches, highlighting their specific strengths and limitations, and their ongoing evolution. Particular attention is given to the recent transition from predominantly mechanistic disease modeling toward increasingly patient-specific and translational applications. Through this perspective, we aim to emphasize that no single model can fully recapitulate the complexity of NDs and that the selection and integration of complementary models should be guided by the specific biological or translational question being addressed.

## 2. In Vitro Models of Neurodegeneration: Past, Present and Future Directions

Compared with other diseases, NDs are particularly challenging to study because human nervous system tissue cannot be readily sampled from living patients, unlike many other tissues and organs. Moreover, as mentioned before, a key insight from NDs research is the profound interconnectedness among disease processes, which manifests in the nervous system’s complex cellular architecture. This architecture underpins our cognitive abilities and disease susceptibility, which represent emergent properties of this biological system and are difficult to mimic, study and understand. At the core of the architecture of the nervous system, cells emerge as the fundamental units, with their unique properties and collective interactions. While the basic architecture appears to be conserved across mammals, various studies have highlighted significant differences in the cellular composition of the human nervous system compared with other species [10,11]. Specifically, marked variations were observed in the proportion of neuronal and non-neuronal cells, and in the transcription of genes associated with neuronal structure and activity [12].

**Figure 1:**
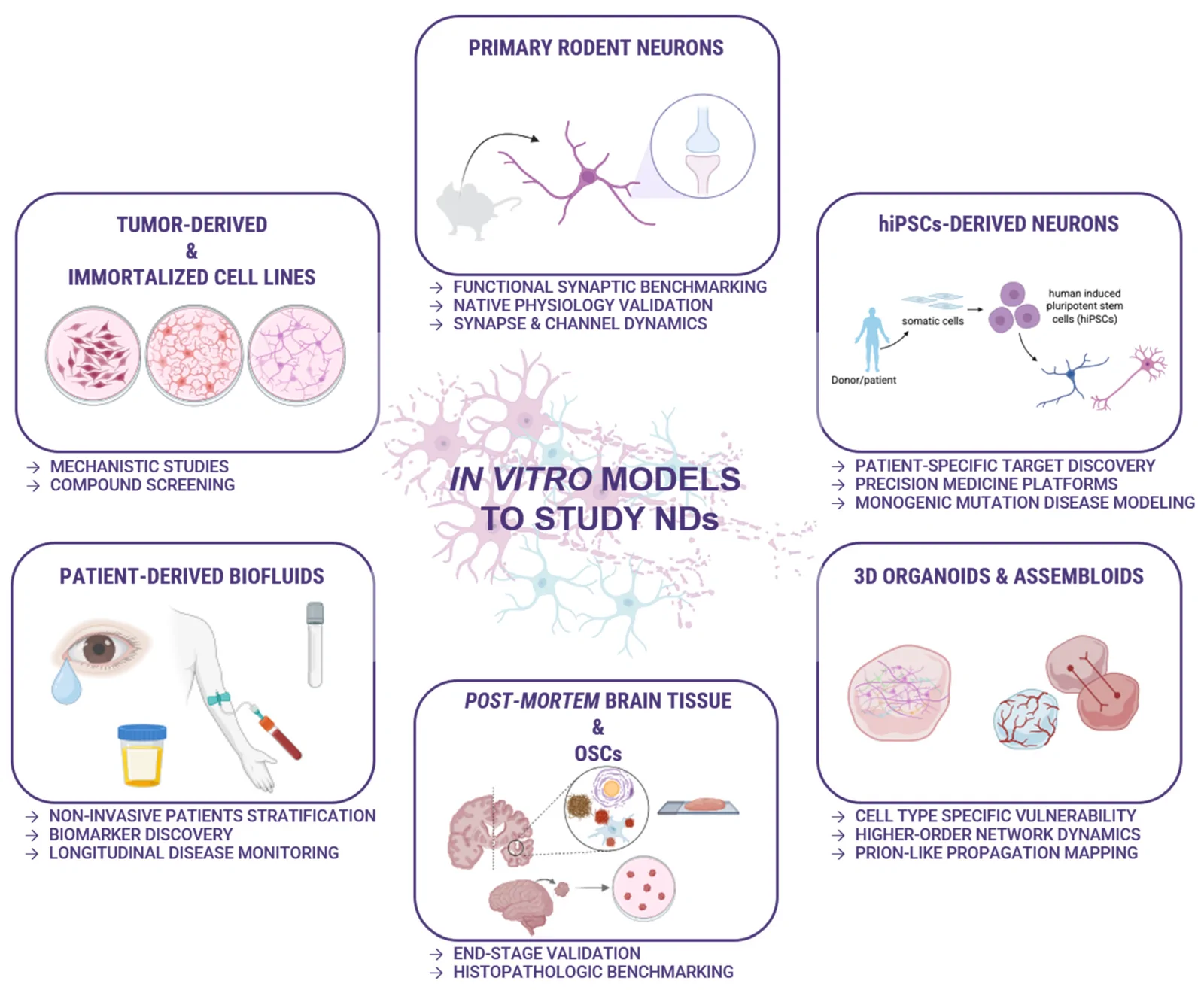
Overview of in vitro models in ND research. Schematic representation of current experimental in vitro models, ranging from 2D cellular cultures to complex 3D tissue architectures, human post-mortem samples, and patient biofluids. Arrows (→) highlight key primary applications, primary diagnostic uses, and clinical utility across platforms.

Several in vitro approaches have been developed over the years to understand the yet unclear etiology and pathogenesis of NDs. Key in vitro models of NDs (Figure 1) include bidimensional neuronal cultures (e.g., immortalized/tumor cell lines and stem cell lines), rodent primary neurons, human-induced pluripotent stem cells (hiPSCs)-derived patient-specific neurons, 3D neural systems (organoids and assembloids), and patient-derived samples (e.g., post-mortem brain tissue, organotypic slice cultures and biofluids).

### 2.1. Bidimensional Neuronal Cultures to Mimic Disease Mechanisms: Tumor-Derived and Immortalized Cell Lines

The most widely used cellular models to study NDs are tumor-derived or immortalized cell lines. Human neuronal tumor cell lines originate directly from malignant human neural tumors (typically neuroblastomas), retaining cancer-associated genetic variants, chromosomal instability, and oncogenic pathways that drive uncontrolled proliferation. In contrast, human immortalized neuronal cell lines originate from non-cancerous primary cells via targeted immortalization (e.g., expression of hTERT and HPV E6/E7), preserving closer-to-native genotypes while conferring indefinite replicative potential.

Tumor/immortalized cell lines are commercially available, easy to culture, and low-maintenance models, offering translational value through their human genetic background [13]. Additionally, their clonal nature and continuous proliferation enable robust reproducibility, making them highly suitable for dissecting fundamental disease-related biological processes through mechanistic studies, albeit with potential emerging genetic aberrations compared with primary cells. These features also facilitate high-throughput screening to evaluate the efficacy, toxicity, or neuroprotective properties of diverse chemical compounds and biological agents [14]. Furthermore, these platforms are widely utilized to establish exogenous stress—such as exposure to neurotoxic insults, mitochondrial complex inhibitors, or misfolded protein species—to acutely recapitulate core pathological hallmarks, including protein aggregation, oxidative stress, and cell death [15,16]. Immortalized cell lines are readily available for major NDs like AD and PD, but gaps exist for rarer disorders, such as particular subtypes of dementias or ataxias, which often lack dedicated immortalized lines [1,17]. The most common immortalized/tumor human-derived cell lines available for modeling NDs are listed in Table 1.

Among the available tumor cell lines, human neuroblastoma-derived SH-SY5Y cells are widely used to mimic some ND stages and can also be differentiated into dopaminergic cells or other neuronal types. Thus, they have been frequently employed to model PD via dopaminergic differentiation, or AD to assess amyloid/tau toxicity [44,45,46,47]. Beyond these specific applications, SH-SY5Y provides a versatile platform for studying mitochondrial dysfunction, a pathological hallmark shared not only by major NDs but also by ALS and HD [48,49]. By modeling dopamine (DA) dyshomeostasis upon DA supplementation, this cellular model further extends its utility to the study of early-stage PD pathogenesis [50]. Moreover, SH-SY5Y cells are a relevant cellular model in the assessment of cytotoxicity for central nervous system (CNS)-targeted drug candidates [51], or in the testing of neuro-toxicants and neuro-disruptors, especially if integrated into blood–brain barrier (BBB) models to assess drug permeability [52]. The immortalized Lund human mesencephalic (LUHMES) cell line mirrors SH-SY5Y (neurotoxicity, mitochondrial assays, protein aggregation) but with enhanced dopaminergic neuron fidelity, making it the gold standard for PD modeling [1].

Beyond classical phenotype-committed immortalized neuronal models, immortalized neural stem cell lines offer multipotent alternatives. HB1.F3 cells (immortalized human neural stem cells from fetal telencephalon) are a multipotent line that can differentiate into neurons, astrocytes, and oligodendrocytes, representing a valid pre-clinical cell therapy platform [34]. Its engineered BDNF variant (i.e., HB1.F3.BDNF) specifically differentiates into medium spiny neurons (MSNs) for HD modeling, complementing classical immortalized neuronal lines [34]. Derived from fetal cortical tissue, CTX0E03 neural stem cells mirror HB1.F3 multipotency but predominantly express cortical phenotypes; furthermore, their clinical-grade, GMP-compliant status ensures safety for direct translation into clinical trials [53]. Other immortalized neural stem cells are the HNSC.100 (human neural stem cells) and the ReNcell VM (human fetal ventral mesencephalic progenitors, *v-myc* immortalized), with the latter being more physiologically relevant for PD modeling as already patterned toward a midbrain identity. In addition to human models, several classic rodent-derived immortalized cell lines remain widely utilized for preliminary screening and basic mechanistic studies due to their rapid proliferation and low cost. These include tumor-derived or somatic hybrid lines such as PC12 (rat pheochromocytoma) and MN9D (mouse midbrain hybrid) for catecholaminergic and dopaminergic toxicity [54,55]; Neuro-2a (N2a) and HT22 (mouse neuroblastoma and hippocampal lines, respectively) for dissecting amyloid processing, Tau pathology, and glutamate-induced oxidative stress; and BV-2 (immortalized mouse microglia) for neuroinflammatory signaling [56,57]. However, while highly accessible, these rodent models carry inherent limitations—including interspecies genomic differences, neoplastic metabolic alterations, and lack of human-specific transcriptomic identity—leading the field to increasingly favor human-derived platforms. These models represent a useful playground for generating hypotheses about disease mechanisms that can be subsequently tested in more complex model systems.

### 2.2. In-Depth Characterization of the Cell Type Mainly Affected in NDs: Primary Neuronal Cultures

Primary cultures partially address key limitations of immortalized cell lines in ND research by maintaining physiological neuronal characteristics, including OXPHOS-dominant metabolism, mature electrophysiological properties with functional synapses and network activity, and genetic stability free from karyotype aberrations [58]. However, as mentioned, isolating human primary neurons remains unfeasible. Researchers thus employ embryonic murine brain tissue as an accessible alternative. To maximize the translational value of primary murine preparations, model selection must be strictly aligned with the specific neuronal subtype and pathological mechanism under investigation. Hippocampal preparations serve as the premier choice when evaluating early synaptic failure, dendritic spine remodeling, and long-term potentiation (LTP) deficits. For broader network-level dysfunction, cortical excitation–inhibition imbalances, and τ transmission, embryonic cortical neurons provide dense, electrophysiologically active networks suitable for long-term multielectrode array (MEA) recording [59]. In contrast, evaluating specific dopaminergic vulnerability—such as susceptibility to protein aggregates, oxidative damage, or mitochondrial toxins (e.g., MPP^+^, rotenone)—requires embryonic ventral mesencephalic (VM) preparations, which enrich for tyrosine hydroxylase-positive (TH^+^) neurons to study catecholaminergic signaling and metabolic stress [60]. While glial contamination occurs, this represents an advantage: mixed neuron-glia co-cultures better recapitulate the multicellular CNS microenvironment influencing neuronal function, survival, and pathology across NDs [61]. For instance, these cultures support key assays for prion-like protein spreading, including α-synuclein (α-syn) preformed fibrils (PFF) seeding for PD propagation and τ fibrillization assays for AD pathology transmission [62,63,64]. Murine primary neurons closely resemble human neurons in morphology, physiology, and post-mitotic nature, but species-specific differences persist (e.g., ion channel patterns and subtype expression) [65]. Primary rodent neurons remain the gold standard for dendritic spine morphology studies due to their physiological maturity and in vivo-like dynamics, critical for understanding synaptic loss in ND progression [66,67]. Recently, a rat-derived neuron-astrocyte-microglia tri-culture model has allowed researchers to more faithfully replicate neuroinflammatory responses, overcoming traditional microglial culturing limitations (short-term cultures, absent cellular interactions, etc.) and opening new pathways for advanced neurotoxicity testing [68]. However, primary rodent culture preparations suffer from high variability due to factors like embryonic age and isolation methods, while long-term maintenance remains challenging, typically limited to 2–4 weeks owing to glial overgrowth and metabolic exhaustion [69,70]. Furthermore, because primary neurons are harvested from embryonic or early postnatal tissue, they inherently represent a developmentally young phenotype that lacks the accumulated epigenetic, mitochondrial, and proteostatic hallmarks of cellular aging—a fundamental prerequisite for modeling late-onset neurodegenerative disorders. Additionally, ethical considerations regarding animal welfare and the high consumption of pregnant dams required for continuous primary dissections create strong pressure to adhere to the 3Rs principles (Replacement, Reduction, and Refinement) [71].

Despite all the advantages of primary cultures, employing primary murine/rodent systems to model human NDs exposes a critical translational gap. The most critical drawback is the persistent interspecies variability and the poor translatability of non-human findings. To circumvent this persistent translational barrier, researchers have increasingly turned toward human-derived alternatives.

### 2.3. The Promise of Pluripotency: Patient-Specific Neurons Derived from Induced Pluripotent Stem Cells

Human-induced pluripotent stem cells (hiPSCs) are pluripotent cells obtained by reprogramming patient-derived somatic cells, including fibroblasts and peripheral blood mononuclear cells (PBMCs), using a variety of established methods, thereby providing an accessible and minimally invasive source of patient-specific cells [72]. These cells can differentiate into virtually any human cell type, providing a powerful platform for generating patient-specific neurons and microenvironment in vitro while overcoming the limited accessibility of primary neural tissue.

Patient-specific hiPSC-derived cells represent a modeling strategy that yields authentic human neurons and glia while preserving the patient-specific genetic background, which is useful for modeling both monogenic variants and sporadic ND forms [73]. Plus, patient-derived hiPSC models serve as powerful tool to uncover disease-specific genetic signatures, identifying novel prognostic biomarkers and druggable therapeutic targets. For instance, PD patient-derived hiPSC neurons with SNCA mutations display specific nitrosative signatures leading to S-nitrosylation of TCA cycle enzymes and consequent mitochondrial metabolic dysfunction [56]. Identifying these target genes and metabolic signatures enables phenotypic high-throughput drug screening; compound libraries can be rapidly screened in patient-derived neuronal models to identify small molecules that rescue mitochondrial membrane potential, promote clearance of toxic protein aggregates, or restore neuronal survival markers prior to clinical translation. These cells bypass ethical issues and enable autologous modeling of the disease together with direct assessment of therapeutic outcomes in the same patient. Deriving cells directly from the recipient makes disease modeling both more reliable and, for cell-based therapies, highly compatible with the same patient, reducing the risks associated with allogeneic stem cell transplants [17]. hiPSCs self-renewal capacity and multi-lineage differentiation potential further enable the study of susceptibility alleles, facilitating the identification of disease hallmarks and clinical subtypes in NDs, though they require broad patients/controls panels for robust phenotype validation [74].

In addition to inefficient somatic cell reprogramming, conventional hiPSC differentiation protocols often suffer from variable neuronal differentiation yield, off-target cell types, poor inter-batch reproducibility, and high operational costs. These technical constraints translate into heterogeneous datasets, limiting experimental scalability and cross-study generalizability [75]. From an analytical perspective, this heterogeneity compromises bulk multi-omics profiling due to the underrepresentation of target neurons, whereas single-cell approaches have proved essential for delivering interpretable, cell-type-specific insights. However, considerable efforts toward protocol optimization and standardization have partially addressed these limitations, leading to improved reprogramming efficiency, more reproducible neuronal differentiation, and greater consistency across independent studies. In particular, the neuroscience community has increasingly relied on centralized and specialized repositories. International biobanks, such as the European Bank for induced pluripotent Stem Cells (EBiSC), now provide highly standardized, quality-control-checked patient-derived and control hiPSCs [76]. These repositories have streamlined the distribution of lines and the generation of isogenic pairs via gene editing, allowing researchers to study specific pathogenic mutations on an identical genetic background, thereby mitigating the confounding effects of donor-to-donor polymorphism.

The implementation of these standardized human platforms has yielded critical mechanistic insights across various NDs, demonstrating the specific biological advantages of human-derived cells. By grafting hiPSC-derived cortical neurons directly into the brain of amyloid precursor protein (APP) transgenic mice, recent studies have successfully tracked the precise, in vivo spatio-temporal dynamics of early AD pathology. This combined approach revealed that extracellular amyloid-β (Aβ) plaques induce highly localized pre-synaptic toxicity and dystrophic neurites strictly restricted to the immediate plaque microenvironment, rather than causing widespread degeneration. Consequently, this human-mouse platform demonstrated that extracellular Aβ exerts an impactful, yet spatially compartmentalized, toxic effect on human pre-synapses [77].

On the other hand, reprogramming has critical limitations that undermine ND modeling fidelity. Extended in vitro culture of hiPSCs can induce genomic instability, while the diverse genetic background of different donors introduces significant line-to-line variability, often confounding experimental outcomes. Moreover, other donor-related factors, such as age, sex, ancestry, and X-chromosome inactivation (XCI) erosion, further compromise physiological accuracy [78,79]. Particularly in female hiPSC lines, XCI erosion during reprogramming causes widespread X-linked gene derepression, creating dosage imbalances that skew neuronal gene expression and synaptic function [80]. Free of this complexity, male lines are preferentially selected as models. This practice perpetuates sex disparities in ND understanding while neglecting female-specific vulnerabilities that drive accelerated disease progression. Furthermore, neuroscience research systematically underrepresents female donors, with female-derived lines comprising less than 30% of ND modeling cohorts. This sex bias represents a profound epidemiological mismatch; indeed, an example is the global burden data estimation of a female-to-male prevalence ratio of 1.69 for AD and related dementias [81]. Plus, reprogramming fibroblasts from aged donors, essential for modeling late-onset PD/AD or later-diagnosed MS, is highly inefficient and erases key disease epigenetic hallmarks. By resetting the epigenetic clock to an embryonic-like state, pluripotency erases age-associated hallmarks (e.g., heterochromatin loss, nucleocytoplasmic transport defects, and mitochondrial decay) required to model late-onset NDs.

Despite these significant technical and biological challenges, hiPSC technology has successfully achieved bench-to-bedside translation. A landmark phase I/II trial successfully demonstrated the long-term safety and therapeutic efficacy of allogeneic iPSC-derived dopaminergic progenitors transplanted into the brains of seven PD patients [82]. Over a 24-month post-transplantation period, the grafted cells demonstrated robust survival and functional DA production, significantly ameliorating motor symptoms without triggering immune rejection or tumorigenic transformation.

As already highlighted for immortalized cell lines, while numerous hiPSC lines have been developed for major NDs (e.g., PD and AD), more complex or rare NDs, such as ALS, pose greater challenges, where polygenic bases and no consistent disease signatures hinder reliable hiPSC-derived model development [83]. Seminal work by Workman and colleagues [83] established the largest hiPSC motor neuron dataset for community-wide omics analysis, biomarker discovery, and modeling standardization, revealing heterogeneous gene expression and sex-specific insights but no universal biomarkers. Conversely, hiPSC-derived neuron models are highly effective for early neurotoxicity screening, providing robust platforms for assessing drug candidate safety and identifying potential neurotoxic liabilities [84]. However, standard 2D monolayer cultures lack native 3D extracellular matrix architecture and realistic cell-type stoichiometry, limitations that have driven the field toward 3D organoid platforms.

### 2.4. Mimicking the Complexity of Nervous Tissue Architecture: Three-Dimensional Models

While the in vitro models discussed so far offer key advantages ranging from high-throughput scalability (immortalized lines) and authentic synaptic functional maturity (primary rodent cultures) to human genetic fidelity (patient-derived hiPSCs), none fully capture the complex 3D tissue architecture of the native human brain for precise modeling of NDs. To overcome these spatial constraints, 3D brain organoids and assembloids have emerged as unique platforms, suited for investigating complex multicellular interactions, regional susceptibility, and non-cell-autonomous mechanisms within a physiologically relevant microenvironment.

Organoids are self-organizing 3D structures derived from iPSCs. They mimic the architecture and function of organs under controlled in vitro conditions that recreate ND niches and microenvironments. In ND research, organoids enable the use of patient-derived cells and circumvent ethical and practical barriers to human brain tissue sampling, as well as interspecies incompatibilities with primary rodent neurons [17].

Organoids enable the generation of region-specific models that capture varying neuronal resilience across brain areas. Indeed, different brain areas exhibit unique combinations of neuronal populations, characterized by distinct degrees of vulnerability, and a network of supportive cells. As an example, the rapid loss of midbrain dopaminergic neurons alongside greater cortical neuronal resistance must be recapitulated to understand PD dynamics [39]. Region-specific organoids capture distinct proteostasis, metabolic, and inflammatory responses. For instance, striatal organoids highlight HD chorea-linked circuit dysfunction absent in cortical ones, while cortical organoids replicate layered architectures prone to τ pathology in AD [85,86]. This regional diversification has proven particularly critical in dissecting the early, non-cell-autonomous contributions of macroglia in specific target structures. Indeed, ALS/FTD modeling using organoids derived from patients carrying *C9ORF72* hexanucleotide repeat expansions has unveiled critical presymptomatic pathological hallmarks. Mechanistically, these patient-specific cortical organoid (CO) slice cultures demonstrated that *C9ORF72*-mutated astroglia display early autophagic dysfunction, while deep-layer neurons accumulate dipeptide repeat (DPR) protein poly(GA) and undergo substantial DNA damage alongside nuclear pyknosis. These localized neurodegenerative events could be pharmacologically rescued by the PERK inhibitor GSK2606414, validating these human-derived 3D cultures as a reproducible translational platform to investigate preclinical ALS/FTD mechanisms and screen for novel therapeutic interventions [87]. Conversely, midbrain organoids (MOs) have pioneered the modeling of cell-autonomous mitochondrial dysfunction, selective lipid peroxidation, progressive α-syn aggregation and prion-like spreading in PD. For instance, Mohamed and colleagues [88] confirmed that the increase in α-syn aggregates within human MOs harboring *SNCA* mutations directly correlates with a reduction in dopaminergic neuron numbers. This acute cell type-specific susceptibility underscores the intrinsic selective vulnerability of dopaminergic neurons, driven by the unique bioenergetic demands of their massive axonal arborization, sustained transmembrane Ca^2+^ influx, and the high baseline oxidative stress generated by cytoplasmic DA oxidation. Furthermore, Frattini and colleagues [89] generated MOs from *GBA1*-mutated PD patient-derived iPSCs, recapitulating Lewy body pathology by associating glucocerebrosidase dysfunction and glucosylceramide accumulation with α-syn aggregation in dopaminergic neurons. These studies support the use of patient-derived region-specific 3D models for dissecting genetic drivers of disease vulnerability, thereby enabling mechanistic studies that bridge anatomical specificity to patient-specific ND progression, and eventually the development of personalized therapies.

However, while region-specific organoids excel at modeling localized cellular vulnerabilities, they remain topologically isolated, failing to recapitulate the long-range axonal connectivity and inter-regional crosstalk that drive systemic ND progression. Pathological hallmarks, such as misfolded α-syn or hyperphosphorylated τ, are rarely restricted to their site of origin; instead, they propagate through neural networks via a prion-like spreading mechanism, as already mentioned.

By fusing region-specific, pre-patterned organoids into so-called “assembloids”, distinct neural niches are put together, increasing model complexity to better recapitulate direct interactions among different brain regions. A milestone in this paradigm shift was the development of Midbrain–Striatal–Cortical Organoids (MISCOs), an engineered fusion platform that successfully reconstructed the topography of the human dopaminergic system in vitro. By physically coupling ventral MOs with their native projection targets (the striatum and cortex), MISCOs provide a human platform to study functional dopaminergic innervation, reciprocal circuit formation, and active DA release into target areas [90]. This multi-axial fusion approach is highly versatile, as it can be utilized to model HD by tracking the axonal spread of pathogenic huntingtin aggregates from the striatal compartment to connected neural populations [91]. Furthermore, the modular nature of this approach allows for the incorporation of hindbrain components to map the serotonergic system, or the integration of hippocampal and retinal organoids to reconstruct complex cognitive and visual circuitries [92,93]. Blood–brain barrier (BBB) assembloids represent another relevant model that extends multicellular complexity by integrating vascular endothelial cells, pericytes, and astrocytes [94]. The BBB acts as the brain’s gatekeeper; its integrity safeguards neural tissue by blocking pathogens, toxins, and excessive immune cells while permitting nutrient and oxygen supply and waste clearance. In MS, early BBB disruption driven by proinflammatory cytokines enables immune cell infiltration, perivascular inflammation, demyelination, and axonal damage, sustaining neurodegeneration in progressive phases and establishing the BBB as a key therapeutic target [95].

Besides being advanced systems, organoids and assembloids present some limitations. Their generation incurs high costs from reagent demands and extended culture durations, restricting scalability for high-throughput applications like drug screening. 3D culture platforms still lack functional vascularization, often exhibit incomplete cellular maturation, and do not fully mirror the precise in vivo anatomical architecture. Eventually, establishing reproducible, standardized protocols remains challenging due to variability in differentiation efficiency, culture conditions, and cell sourcing, impeding cross-study and inter-laboratory comparisons [96]. When comparing experimental platforms to select the most appropriate model, a clear trade-off emerges between screening capacity, scalability, structural fidelity, and multicellular complexity. While immortalized cell lines provide scalable platforms for preliminary drug screening and basic mechanistic dissection at the expense of biological fidelity, and primary rodent cultures offer synaptic maturity with interspecies limitations, 3D organoids and assembloids represent the gold standard for modeling human-specific cell–cell crosstalk and inter-regional aggregate propagation. Organoids and assembloids represent an invaluable tool that effectively allows transition from single-cell architecture to the higher-order network dynamics that drive NDs. To comprehensively map these intricate cellular dynamics and validate the structural organization of both isolated organoids and multiregional assembloids, researchers have increasingly relied on the integration of multi-omics approaches and advanced imaging. Conventional bulk transcriptomic or proteomic analyses inherently mask the discrete cellular responses within these heterogeneous tissues, averaging out the unique signatures of rare or highly vulnerable sub-populations. To overcome this limitation, single-cell omics technologies (such as scRNA-seq, snRNA-seq, and spatial omics) have become indispensable tools. When applied to organoids, single-cell sequencing allows researchers to delineate cell-type-specific developmental trajectories with high fidelity, verifying whether stem cell-derived lineages successfully mirror human-specific maturation over months [97]. By applying single-cell multi-omics to complex assembloids, researchers can detect non-cell-autonomous alterations within a healthy recipient organoid, driven exclusively by its physical coupling to a genetically altered or patient-derived donor culture [91].

### 2.5. Beyond the Living Brain: Post-Mortem Tissues, Organotypic Slice Cultures, and Biofluids

Post-mortem samples, particularly patient-derived frozen brain tissues, have long been considered the gold standard for validating key findings obtained from the models described above, especially 2D neuronal cultures. While preserving the native 3D cytoarchitecture and microenvironment, these samples typically capture only end-stage pathology, with limited insight into disease progression [98]. This is particularly important when studying pathologies that develop silently over decades before the onset of clinical symptoms, meaning that the pathological features observed at the final stage do not necessarily reflect the critical events underlying the disease cascade. By the time the tissue is analyzed, the disease has already reached its most advanced stage, characterized by extensive cell death, profound tissue atrophy, and the widespread accumulation of protein aggregates (such as amyloid plaques or τ tangles). Moreover, despite recent international efforts to standardize sample collection and preservation procedures, substantial variability persists in post-mortem delay and tissue integrity, making comparisons across studies challenging and compromising the statistical robustness of validation efforts.

More recently, alongside conventional post-mortem tissues, organotypic slice cultures (OSCs) have gained increasing attention as complementary experimental models, as they retain key features of the tissue architecture while allowing easier manipulation. OSCs are ex vivo tissue preparations in which thin slices derived from fresh neurosurgical tissues are maintained in culture under controlled conditions that preserve native architecture, cellular composition, and cell–cell interactions. Indeed, hiPSC-derived neurons are routinely benchmarked against them for transcriptomic fidelity (e.g., ALS *TDP-43* signatures), confirming physiological relevance despite end-stage biases [99]. However, the translational potential of OSCs faces logistical hurdles, high donor-to-donor variability, and severe difficulties in long-term maintenance. The high vulnerability of fresh adult human tissue and the lack of standardized protocols for tissue collection (e.g., handling temperature, transport conditions, etc.) represent fundamental limitations that heavily influence overall sample quality and the reliability of downstream validation outcomes [100].

By contrast, the use of patient-derived biofluids (e.g., cerebrospinal fluid (CSF), blood, saliva, urine, tears) offers a valuable platform for biomarker discovery, allowing for early diagnosis, prognosis, patient stratification and disease monitoring and modeling [101]. These accessible and less invasive biological samples facilitate early biomarker discovery and verification through longitudinal sampling, which is essential for capturing the dynamic evolution of the pathology [102,103]. Ultimately, the shift from static, terminal tissue analysis to fluid-based longitudinal monitoring is critical for stratifying ND subtypes pre-symptomatically, thereby opening a temporal window for therapeutic intervention that post-mortem analysis simply cannot provide [101]. For instance, alterations in distinct α-syn species have been detected in saliva from patients with PD, supporting the potential of peripheral biofluids to provide disease-relevant molecular signatures even when these do not necessarily recapitulate changes occurring within the CNS [104]. Interestingly, recent literature has identified numerous urinary metabolic signatures associated with PD, involving pathways related to energy and mitochondrial metabolism, oxidative stress, neuroinflammation, and the gut–brain axis. Notably, a systematic review encompassing forty studies of urinary metabolomics in PD highlighted the potential of this approach to capture disease-associated metabolic alterations [105]. These findings underscore how peripheral biofluids can provide indirect insights into systemic pathological processes associated with NDs, even when the identified biomarkers are not directly released from the brain. Indeed, an important consideration regarding the use of biofluids in NDs research and early diagnosis concerns the purpose for which they are employed. Changes detected in the periphery do not necessarily mirror those occurring in the CNS, and vice versa. Rather, biofluid analysis is particularly valuable because it can provide insights into disease mechanisms and reveal early biomarkers that may not be detectable when investigations are restricted to the CNS.

To provide a comprehensive decision-making framework for selecting the most appropriate in vitro model in NDs research, Table 2 summarizes the key biological and technical features of all reviewed platforms, highlighting their complementary roles in neurodegenerative disease research. Specifically, in vitro models serve as the gold standard for high-throughput screening, dissecting human cell-autonomous mechanisms, and precise molecular target discovery, whereas transitioning to in vivo animal models becomes necessary when investigating whole-organism physiology, long-term chronic progression across aging, multi-systemic interactions, or complex behavioral and cognitive outcomes.

## 3. In Vivo Models: Capturing Emergent Properties at the Organism Level

As already mentioned, in vitro models fail to recapitulate the full spectrum of disease pathogeneses because of their simplistic structure [106], and they cannot provide organism-level functional and behavioral responses, including complex inter-organ interactions [107,108]. Therefore, in vivo models are fundamental for studying NDs as whole-body disorders by capturing brain–body interactions involving neuroinflammation and immune response, metabolic disturbances, the gut-brain axis, and peripheral organ involvement, modulated by aging and its associated comorbidities [109,110,111]. Furthermore, they enable the assessment of behavioral phenotypes and cognitive decline that cannot be recapitulated in vitro [107,112,113]. Accordingly, animal models are widely used in preclinical research on NDs [114]. The translational value of animal models is commonly defined by three criteria: face (surface-level appearance), construct (theoretical alignment), and predictive (future forecasting) validity, which help determine whether a model can recapitulate key physiological and pathological features of diseases [71,72,73]. The choice of the experimental species is driven by the scientific aims of the study and considers biological, anatomical, functional and genetic similarities to humans [115].

**Figure 2:**
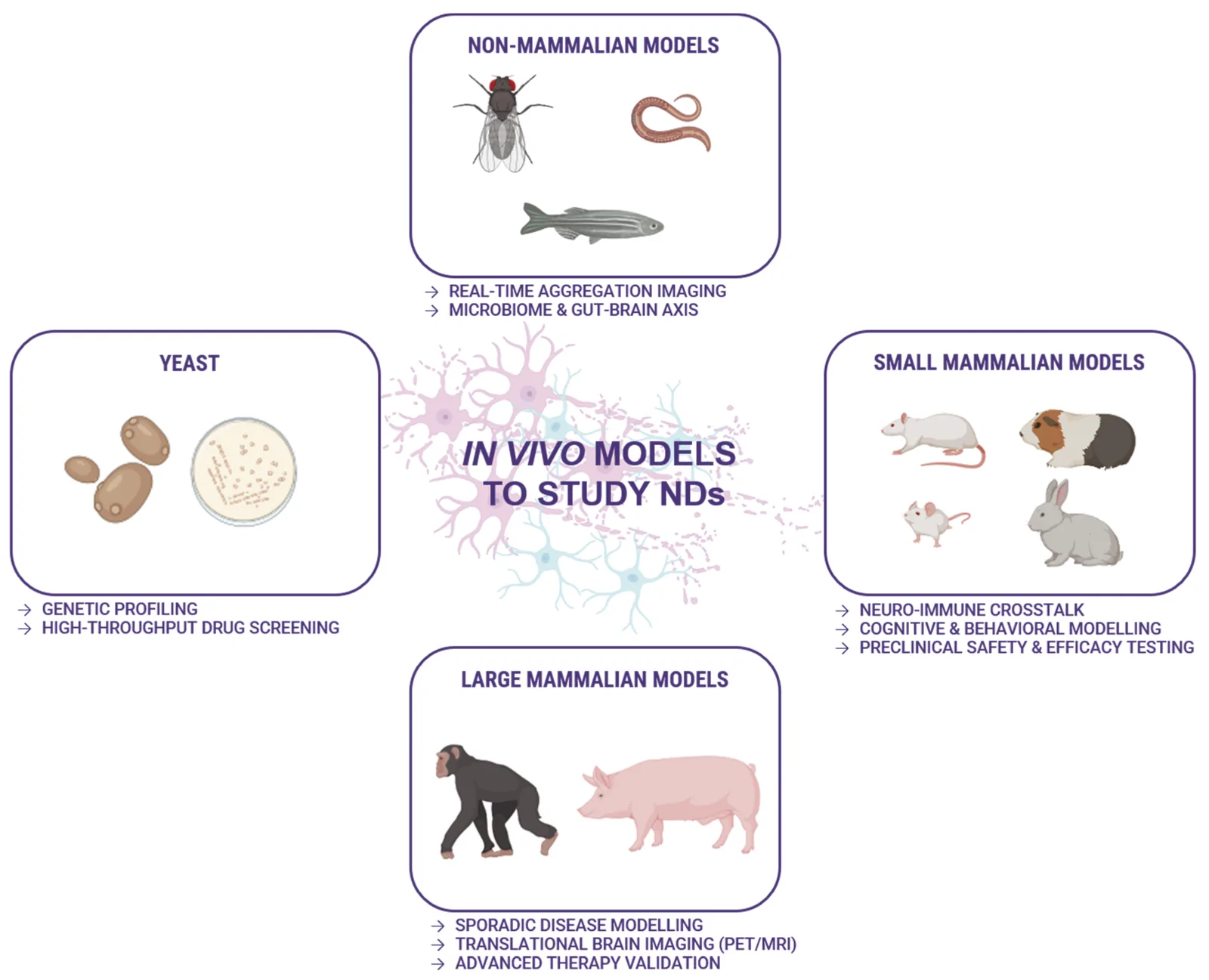
Overview of in vivo experimental models in NDs research. Schematic representation of model systems across biological complexity, ranging from unicellular yeast (*S. cerevisiae*) and non-mammalian organisms (*D. melanogaster*, *C. elegans*, *D. rerio*) to small (mouse, rat, guinea pig, rabbit) and large mammalian models (non-human primates, pigs). Arrows (→) highlight primary applications.

In this section, we describe the most widely used in vivo models with progressively increasing biological complexity, discussing their applications, advantages and disadvantages, and translation value: yeast, non-mammalian model organisms, small mammals, and large mammalian animals (Figure 2 and Table 3).

### 3.1. Studying Evolutionarily Conserved Disease Mechanisms: Yeast Models

Yeast represents the simplest eukaryotic organism and is particularly suitable for the investigation of evolutionarily conserved biological pathways in both physiological and pathological conditions [118]. The success of this unicellular organism as a disease model is associated with the high efficiency of the homologous recombination mechanism, which enables the rapid generation of genetic knockouts, and its ease of manipulation [119].

The value of *S. cerevisiae* as a model extends beyond practical convenience. Indeed, this organism has long served as a pioneering model for the study of fundamental eukaryotic cellular processes. Landmark discoveries in cell cycle regulation, apoptosis and mitochondrial biology were first made in yeast, and the underlying molecular mechanisms were subsequently found to be conserved in higher eukaryotic species, including humans. Accordingly, many genes encoding proteins involved in these pathways were first identified and characterized in *S. cerevisiae*, with the function of their human orthologues only later elucidated [119,120].

Based on these considerations, yeast has proven a valuable tool for the study of ND-related genes, where conserved pathological mechanisms between *S. cerevisiae* and humans can be directly exploited. Over the last two decades, yeast cells expressing human amyloidogenic proteins have been developed, becoming crucial tools for unveiling key components involved in the metabolism and toxicity of these proteins [121]. In the context of AD, this model contributed to the investigation of Aβ protein dysfunction and mitochondrial protein processing. Notably, Brunetti and colleagues validated the pathogenicity of a missense mutation in *PITRM1*, a mitochondrial matrix metallopeptidase responsible for the degradation of Aβ within mitochondria. This study demonstrated that partial loss of PITRM1 protease function leads to impaired mitochondrial Aβ clearance and enhanced Aβ accumulation, providing evidence for a direct mechanistic link between mitochondrial peptidase dysfunction and AD [122,123].

Another productive application of this model was its use as a platform for high-throughput drug screening. Exploiting the conservation of ubiquitin-mediated degradation pathways, a fluorometric yeast-based screening system demonstrated high sensitivity and specificity in identifying compounds that suppress α-syn aggregation or enhance its ubiquitin-dependent clearance, highlighting the potential of this model for PD drug discovery [117].

Despite several advantages of yeast as an experimental model, important constraints should be highlighted. Indeed, yeast cannot be used to model diseases at the level of organs or whole multicellular organisms, precluding the investigation of tissue-specific effects and disease progression in an integrated physiological context [118,143]. Nevertheless, yeast provides strong construct validity through the conservation of fundamental cellular pathways, while its unicellular nature limits face and predictive validity. On the other hand, yeast is best regarded as a powerful platform for in-depth characterization of gene functions and protein-protein interactions, rather than as a comprehensive disease model.

### 3.2. Studying NDs at the Whole-Organism Level: Non-Mammalian Model Organisms

Non-mammalian model organisms constitute a higher-complexity tool for studying NDs. Commonly used animals include flies (*D. melanogaster*), worms (*C. elegans*) and fishes (*D. rerio*, zebrafish). These organisms are easy to maintain, cost-effective, exhibit rapid life cycles and can be easily monitored throughout treatment duration. In addition, they are well suited for biomarker analysis, evaluation of putative drug candidates and mechanistic studies [114]. Although the direct translational potential to humans is limited, these animal models offer the advantage of recapitulating the effects of pathogenic processes at the whole-organism level.

*D. melanogaster* is an invertebrate model characterized by minimal genetic redundancy and a high degree of genetic orthology with mammalian species. Its nervous system displays a well-organized structure with a moderate complexity compared with the human brain [144]. This model is widely used to investigate the interplay between the immune and nervous systems. Consistent with this, a Drosophila model expressing human α-syn in dopaminergic neurons showed accelerated activation of innate immunity pathways, including processes resembling neutrophil degranulation, platelet activation and NF-κB signaling, which have also been implicated in human PD [145]. Importantly, *D. melanogaster* also enables behavioral assessment of motor dysfunction through quantitative assays of locomotor performance, including climbing and flight duration, providing functional readouts of disease-associated motor phenotypes and reflecting some degree of face validity [124].

Both *C. elegans* and zebrafish larvae are considered robust models to mimic ND phenotypes and to facilitate drug screening.

*C. elegans* has historically served as a valuable model organism for investigating the mechanisms underlying NDs, particularly in the context of PD. This organism possesses eight well-mapped dopaminergic neurons [146], which, combined with its transparent body, allows in vivo visualization of ND processes in real time [147]. This model has been widely employed to investigate disease-relevant mechanisms, including protein aggregation, DA depletion, and neuronal loss, primarily through the overexpression of human *SNCA* and *LRRK2* [126,148]. Its utility is further enhanced by the assessment of functional and behavioral readouts, such as locomotor dysfunction, providing an additional layer of phenotypic characterization [126].

Conversely, zebrafish PD models are usually generated using the neurotoxin 1-Methyl-4-phenyl-1,2,3,6-tetrahydropyridine (MPTP) to induce dopaminergic neuron loss and PD-associated locomotor phenotypes similar to the ones found in human PD patients [149]. In particular, zebrafish, due to its conserved gastrointestinal and nervous systems, has recently emerged as a valuable platform for investigating gut-brain axis (GBA) dysfunctions associated with neurotoxicity and exploring microbiome-mediated mechanisms relevant to neurodegeneration [150,151]. Despite the complexity and individual variability of the microbiome, a growing body of evidence supports a mechanistic role for microbial factors in PD pathogenesis. Interestingly, certain gut bacteria, such as curli-producing *E. coli*, express amyloidogenic proteins that can cross-seed with endogenous α-syn, promoting its misfolding and oligomerization in the enteric nervous system, as α-syn aggregates are present in the gut of patients with PD [125]. According to the Braak hypothesis, these enteric aggregates may then propagate retrogradely to the brain via the vagus nerve in a prion-like manner, providing a plausible link between gut dysbiosis and central dopaminergic degeneration. In this view, zebrafish represents a powerful tool for future studies aimed at elucidating α-syn aggregation and spreading in PD pathology [152,153].

Despite advantages, the main limitation of these animal models is related to their simpler nervous system compared with humans, which does not allow for the acquisition of complex information and the direct translation to the human brain [114,115]. Besides, predictive validity remains limited by species-specific differences in neurobiology, pharmacokinetics and drug responses. Thus, these models are mostly valuable for elucidating conserved disease mechanisms and prioritizing candidate compounds before validation in mammalian systems.

### 3.3. Modeling Disease Mechanisms and Therapeutic Responses: Small Mammalian Models

Mammalian models are essential for studying NDs because, despite differences in scale and complexity, the overall organization of the CNS (cortical layering, major neurotransmitter systems, and glial cell populations) is broadly conserved across species, allowing researchers to model neuronal loss, protein aggregation, and circuit dysfunction. At the same time, each species offers a different trade-off between experimental tractability and similarity to humans [154]. The most common mammals used for studying neurodegeneration are mice and rats, while rabbits and guinea pigs are employed less frequently [114]. These ND models are generated either through transgenic techniques, which enable the expression or disruption of genes associated with disease, or through toxin-based approaches, which reproduce specific aspects of neurodegeneration without genetic modification [155,156]. Among in vivo models, small mammals provide a broad translational profile. Genetically modified rodents can closely reproduce disease-associated genetic alterations, whereas toxin- and lesion-based models recapitulate specific neuropathological and behavioral phenotypes, providing both face and construct validity. However, pharmacological responses in rodents do not always translate to clinical efficacy in humans, highlighting the need to interpret preclinical findings alongside evidence from complementary experimental systems.

Murine models are essential tools for dissecting disease mechanisms and investigating immune–nervous system interactions, particularly the role of microglia in neurodegeneration and the therapeutic potential of immunomodulation [157]. While mice are used preferentially when advanced genetic manipulations are required, rats are valuable in specific experimental settings as they better recapitulate age-related neurodegeneration and cognitive decline, for studying the natural progression of NDs in an aging brain [158].

Transgenic mice have been developed and employed to study different NDs, such as AD, PD, and HD. They have been used to elucidate prion-like cell-to-cell transmission of misfolded proteins, such as α-syn and τ proteins [127,128,129], but also for preclinical drug screening, allowing researchers to evaluate therapeutic efficacy and safety before translation to humans [159,160]. However, transgenic mice could also be used to understand disease mechanisms and discover new therapeutic targets. Leitão and coworkers generated an APP transgenic mouse to model AD, and they explored the pathological and behavioral phenotypes by silencing α-syn through RNA interference. Their results suggest a reduction in Aβ plaque burden and propose a novel link between Aβ and α-syn [161].

Historically, transgenic mouse models have been used to demonstrate that disease-associated mutant proteins drive the pathological and behavioral features of NDs [162,163]. More recently, these models have allowed researchers to investigate molecular pathways linking protein aggregation to downstream neurodegenerative mechanisms. In PD, Geng and colleagues generated α-syn transgenic mice with knockout of mixed-lineage kinase domain-like protein (MLKL) to understand the impact of MLKL deficiency on disease progression. Although the precise role of MLKL in PD remains unclear, their results show that MLKL deficiency improves motor function, reduces phosphorylated α-syn expression, and attenuates neuroinflammation [131]. Similarly, in HD, Zinter and coworkers used R6/1 transgenic mice to study the role of retinoic acid receptor beta (RARβ) dysregulation, demonstrating that genetically impaired RARβ signaling accelerates the onset of motor abnormalities and enhances disease progression and aging, whereas normal RARβ expression delays the course of HD [164]. Looking ahead, pathways such as MLKL-mediated necroptosis and RARβ signaling represent promising therapeutic targets for further investigation, and transgenic models are likely to play an increasingly important role in validating candidate interventions aimed at reducing neuroinflammation and slowing disease progression in PD and HD.

Rats are preferentially used for behavioral and sensorimotor tests as they show more complex cognition, are easier to train and show more robust performance in tasks such as maze navigation, object recognition and gait analysis [132,165,166,167]. Behavioral studies can be used to evaluate the positive or negative effects of administered molecules on cognitive changes. As an example, a study by Blossom and coworkers aimed at investigating the role of pioglitazone in oxidative stress and cognitive changes in a rat model of LPS-induced neuroinflammation. They used behavioral tests to examine cognitive differences between the experimental groups (control and treated rats). Through the Morris water maze test, they observed a significant impairment in learning and memory in LPS-exposed animals, whereas pioglitazone-treated groups showed improved cognitive performance [132].

Rats are also used for studying neuroinflammation and microglial activation in different NDs, allowing researchers to investigate the interactions between immune and nervous systems [132,133]. TgF344-AD rats were used to study the neuro-immune crosstalk in AD, showing hippocampal neuronal and astrocytic activation, microglial phenotype, Aβ clearance and striatal dopaminergic signaling [133].

Genetically modified rodents also provide a methodological bridge between in vitro and in vivo approaches. Primary neuronal and glial cultures derived from transgenic or knock-in animals retain the disease-relevant genetic background of the parental model while offering the experimental tractability and reproducibility of in vitro systems. Such cultures can be used to investigate synaptic dysfunction, protein aggregation, oxidative stress, and neuronal viability and to screen candidate compounds before progression to more complex in vivo models [168].

More recently, rats were used as in vivo hosts for human brain organoids, allowing researchers to study neural repair and specific aspects of neural circuit function in a living, physiologically intact system. This xenotransplantation strategy overcomes a major limitation of conventional brain organoid cultures, which, despite recapitulating aspects of human cortical development and cytoarchitecture, generally fail to mature fully or to establish functional and long-range connections with sensory and behavioral circuits in vitro. By providing an active host environment, this system supports organoid maturation, synaptic integration, and circuit-level functionality, opening up new strategies for studying NDs [169]. Revah and colleagues recently proposed a novel approach for detecting circuit-level phenotypes in patient-derived cells. They demonstrated that human stem cell-derived cortical organoids, when transplanted into the somatosensory cortex of newborn athymic rats, can develop mature cell types, including functional neurons and glia, that structurally and functionally integrate into host sensory and motivation-related circuits. Transplanted neurons extended axons over long distances, received sensory inputs, and influenced rats’ behavior [169]. Such models provide a further experimental bridge between human cellular systems and whole-organism physiology, as they allow patient-derived cells carrying disease-relevant mutations to be studied within an intact, functioning neural circuit to investigate how ND-associated pathology (e.g., protein aggregation, synaptic dysfunction, or selective neuronal vulnerability) affects the whole system. This supports the rat-organoid chimera as a powerful platform for linking molecular and cellular pathology to systems-level dysfunction in PD, HD, and other NDs.

Rabbits and guinea pigs are used less frequently than mice and rats for studying NDs and are typically reserved for modeling specific disease phenotypes or mechanistic aspects. Practical limitations exist, including longer breeding times and higher housing costs compared with conventional rodent models. Additionally, their relatively short lifespan (2–3 years) still limits the full development of age-related pathological hallmarks observed in humans [170]. Further limitations arise from genomic and physiological differences compared with humans, particularly in CNS maturation and anatomy, which include a lower white-to-grey matter ratio and the absence of cortical gyrification [171]. This can influence, for example, how amyloid and τ pathology develop [172].

Rabbits are phylogenetically closer to humans compared with mice, and they exhibit greater similarities in brain development. However, there are very few rabbit models for studying NDs, largely because standardized and validated behavioral, cognitive, and biomarker assessment tools have not yet been established, unlike the well-characterized tests available for rodents [173]. Brooks and colleagues used cholesterol-fed rabbits to study the effect of hypercholesterolemia as an increased risk factor for developing AD. These models exhibited brain changes similar to those observed in patients, providing important insights into possible mechanisms involved in the development of the disease and shedding light on Aβ and τ phosphorylation changes hypothesized to cause AD symptoms and pathology [134]. Rabbit models have also been used for studying and evaluating neurotoxicity. Researchers used formaldehyde (FA) to induce toxicity and study the effects of acute oral exposure to this chemical compound in rabbit brain. They observed that FA-induced toxicity caused DNA damage, promoted apoptosis, and induced neuronal injury [174].

Guinea pigs are used in specific and limited contexts in ND research, but they present unique advantages, particularly for studying AD. Notably, their Aβ peptide sequence closely resembles that of humans, unlike mice and rats, where three amino acid substitutions reduce amyloid aggregation propensity. This similarity makes guinea pigs a potentially valuable non-transgenic model for investigating AD [130,175], supporting their broad construct validity. For example, Wahl and colleagues examined two guinea pig strains that appear to naturally mimic key features of human brain aging and AD. Their findings revealed transcriptomic similarities between guinea pig brains and those of aging humans and AD patients. These two strains showed an increased level of neurofilament light chain, Aβ and phosphorylated τ, which are important markers associated with neuroinflammation and AD [130].

Collectively, rabbits and guinea pigs may become increasingly valuable for modeling the progressive development of specific pathological phenotypes over time and features relevant to sporadic forms of NDs. In contrast, murine models will likely remain the gold standard for dissecting disease mechanisms associated with specific mutations in disease genes, as well as for toxicological and therapeutic screening. This potential is further strengthened by the increasing application of advanced neuroimaging approaches in small laboratory mammals, which provide valuable longitudinal and in vivo insights into disease progression and the underlying mechanisms of pathology. Lastly, small mammalian models provide complementary levels of disease relevance, ranging from genetically driven mechanisms to experimentally induced phenotypes. The integration of genetically modified animals, primary cultures, organoid transplantation and advanced neuroimaging further expands the translational potential of these models by enabling disease investigation across molecular, cellular and whole-organism levels.

### 3.4. Bridging Preclinical Models and Human Disease: Large Mammalian Models

Small mammalian animals, as described, have proven to be contributory in advancing our understanding of molecular alterations underlying NDs; however, they fail to reproduce the overt and selective neurodegeneration observed in AD, PD, ALS and HD, strongly limiting their potential to suggest and validate effective disease-modifying interventions [176]. These constraints reflect important interspecies differences in neurodevelopment and brain anatomy. These limitations have prompted growing interest in the use of large animal models, whose brains more closely resemble the human brain, not only in size and architecture but also in connectivity, white matter organization, and gyrification patterns [177,178]. Moreover, large mammals share more comparable aging trajectories and behavioral repertoires with humans, making them particularly suitable to model the progressive nature of NDs and to assess functional outcomes that are clinically relevant [179]. Of all the models discussed in this review, large mammals offer the highest overall translational potential across the three dimensions described above. In this frame, non-human primates (NHPs) and pigs have emerged as the most widely used large animal platforms to model NDs.

NHPs, primarily macaques (*M. mulatta*, *M. fascicularis*) and common marmosets (*Callithrix jacchus*), occupy a unique position among large animal models for NDs. Although experiments in NHPs account for a small percentage of total research in animals, their impact on clinical translation is much greater given their similarity to humans in brain structure and function [180]. From a neuroanatomical perspective, NHPs possess features that increase the complexity of their behavior and cognitive abilities, such as the presence of cortical folding, segmentation and expansions that are more similar to humans [178]. Also at a molecular level, rhesus monkeys show gene expression patterns and developmental trajectories that closely resemble those observed in humans [181]. Their longer lifespan is also a key advantage, as it allows the modeling of age-related ND processes, better mimicking the pathophysiological processes observed in patients. Indeed, NHPs spontaneously develop several age-related neuropathological alterations such as impaired hippocampal neurogenesis, synaptic dysfunction, Aβ deposition and τ hyperphosphorylation, as well as domain-specific cognitive decline, making them particularly relevant for late-onset sporadic NDs [136,137,138]. Moreover, the larger size and functional complexity of NHP brains also enable the implementation of clinically relevant methodologies, including MR- and PET-based neuroimaging, deep brain stimulation (DBS), and optogenetic approaches, which are difficult to reproduce in smaller animals [182,183].

Chemically induced NHP models were among the first approaches developed to reproduce hallmark features of NDs and remain widely employed, as they offer rapid and reproducible induction of disease phenotypes. Classical neurotoxins, namely MPTP and quinolinic acid, have been extensively used to model nigral dopaminergic and striatal degeneration, resembling PD and HD, respectively [184,185]. More recently, intracerebral injection of misfolded proteins, such as Aβ oligomers and α-syn aggregates, has emerged as an insightful strategy to reproduce protein aggregation and neuroinflammation and to investigate spreading mechanisms characteristic of human pathologies [186,187]. The combination of rapid lesion induction with the anatomical and functional similarity of the NHP brain provides a useful intermediate approach between conventional rodent models and human disease.

Recent advances in viral vector delivery systems and genome-editing technologies have enabled the generation of genetically modified NHP models that more faithfully mimic the progressive neuropathological and behavioral aspects of NDs, thereby enhancing their relevance for translational research and preclinical drug development [188]. Interestingly, a macaque model of AD recapitulating widespread τ pathology, neuronal loss, neuroinflammation and multimodal cognitive dysfunction was recently developed [139]. Importantly, unlike rodents, rhesus monkeys express both 3R and 4R τ isoforms, making them particularly suitable to study human tauopathies [139,140]. For PD, Han and coworkers generated a CRISPR/Cas9 parkin-deficient monkey model that developed age-dependent substantia nigra neurodegeneration and pathological α-syn accumulation. Restoration of parkin expression rescued α-syn toxicity, suggesting the therapeutic potential of this approach [189].

In addition to genetic models, NHPs have also proven to be valuable for dissecting the role of GBA across NDs, although this aspect remains most significant for PD research. Compared with rodents, NHP gut microbiota composition and diversity more closely resemble the human gut ecosystem, reinforcing the translational value of ND pathways associated with the microbiome [190]. Accordingly, enteric or striatal injection of pathological protein in baboon monkeys has been shown to trigger bidirectional, gut-to-brain and brain-to-gut propagation of synucleinopathy, consistent with the existence of a structural connection between central and peripheral pathology [191]. More recently, a multi-omics profiling of MPTP-induced parkinsonism in cynomolgus monkeys has linked disrupted gut microbiota-derived metabolites to PD-related pathway dysregulation, highlighting the relevance of host-microbiota interactions to disease pathogenesis [192]. Altogether, these findings support the relevance of NHP models for elucidating gut-brain interactions as key mechanisms in NDs and for the investigation of possible microbiota-related biomarkers and therapeutic targets. Indeed, beyond mechanistic studies, NHPs also provide a valuable platform for preclinical therapeutic evaluation, including assessment of biodistribution and immunogenicity profiles that more closely resemble the human condition [141,142].

Regarding pigs (*Sus scrofa*), they display several complex characteristics in anatomy, physiology and genome that are useful for modeling NDs. There is high similarity between the neurodevelopment and global structure of pig and human brains, specifically at the level of the neocortex, and the white and grey matter densities of porcine brain are comparable to those in humans [193]. Pigs additionally exhibit complex cognitive and social behaviors that enable translational assessment of motor and neuropsychiatric phenotypes in ND research [194]. From a practical perspective, pigs reach sexual maturity early (5–8 months), have a short gestation period (114 days) and usually produce numerous offspring, giving them distinct advantages over NHPs for genetic manipulation through somatic cell nuclear transfer (SCNT) technology [135]. Furthermore, the body size and physiology make these animals a suitable model for drug development and toxicological studies.

As mentioned, the advent of more precise genome-editing tools has facilitated and extended the use of pigs as models for ND studies. Combining CRISPR/Cas9 technology with SCNT now allows the introduction of specific disease-causing mutations directly into the endogenous porcine genome. In a recent study, Wang and colleagues targeted three distinct genomic *loci* simultaneously, *PRKN*, *DJ-1*, and *PINK1*, in Bama miniature pigs, generating a triple gene-targeted pig model of PD in a single step [195]. Not surprisingly, piglets at ten months of age did not display overt PD phenotypes, probably reflecting the long prodromal stage of human disease. However, successful editing was confirmed at a molecular level by reduced mRNA expression of *PRKN* and *PINK1* and loss of DJ-1 protein in fibroblasts, providing a large-animal platform for future longitudinal studies [195,196]. For HD, a knock-in pig model expressing full-length mutant HTT was generated. In this study, characterization of founder and F1knock-in pigs revealed consistent movement and behavioral abnormalities, and germline-transmissible early death. Importantly, brains of these animals showed marked and selective degeneration of medium spiny neurons, a hallmark of HD pathology that had not been previously reproduced in rodents [197]. This pig model was subsequently employed to test gene therapy approaches to reduce mutant HTT expression and neurological symptoms [198]. These results clearly demonstrate that large animal models can reproduce overt neurodegeneration observed in HD patients, highlighting their value to study NDs and their translational potential.

Despite the clear potential, the use of these models is associated with ethical concerns and substantial practical and economic challenges, including high maintenance costs and extended experimental timelines (Table 3). Indeed, large mammalian species require specialized housing, trained personnel and considerable financial resources to ensure proper well-being of animals, while sample size is significantly restricted by the slow reproductive rates of these animals, thus hampering statistical robustness [199,200]. Moreover, unlike inbred rodent strains, pigs and NHPs are outbred species whose genetic background increases phenotypic variability, thus reducing reproducibility across studies [201]. At a technical level, while advantageous, genome editing via CRISPR/Cas9 editing of zygotes in both species frequently generates mosaic mutations or off-target events, which cannot be rapidly resolved through breeding given the long generational intervals [202]. Importantly, some of these limitations can be partially mitigated as longitudinal monitoring can be achieved in a non-invasive way through blood and CSF analysis, including Aβ42, Aβ40, soluble TREM2, IL6 and phosphorylated τ, thereby minimizing the need for terminal procedures [203].

Overall, the use of in vivo models provides a connection between conventional preclinical systems and human disease, allowing the characterization of neural cell types, developmental patterns, organ function, and the effects of aging and pharmacological treatments. However, no single animal model can fully recapitulate the complexity of an intact nervous system and the multifactorial nature of human disease phenotypes. This limitation highlights the need for complementary approaches that capture, model and possibly predict different aspects of disease pathogenesis [4], also in accordance with the adoption of mitigation strategies aligned with the 3Rs principle (Replacement, Reduction, Refinement). In this context, in silico models have recently gained increasing attention.

## 4. In Silico Models: Integrating Biological Knowledge for Predictive Disease Modeling

In silico disease models are emerging as powerful complementary tools for the study of NDs. Compared with traditional experimental approaches, computational models offer several advantages, including reduced costs, shorter development times, high-throughput scalability, and a substantial reduction in animal use. Furthermore, once trained and validated, models can be interrogated under multiple simulated conditions, enabling rapid hypothesis generation and prioritization of experiments.

Recent advances in machine learning (ML) and artificial intelligence (AI) have significantly expanded the scope of in silico disease modeling. Rather than simply reproducing disease-associated features, modern ML-based models aim to learn complex relationships embedded within biological data and to predict the behavior of biological systems under previously unobserved conditions. By integrating information across molecular, cellular, and clinical levels, these models have already been applied to tasks such as biomarker identification, patient stratification, and prediction of disease trajectories, while their use for simulating therapeutic responses or environmental perturbations remains an active area of research [204].

### 4.1. Model Construction: From Data Acquisition to Disease Prediction

The development of predictive models relies on the availability of large and diverse datasets. Multi-omics technologies, including genomics, transcriptomics, proteomics, metabolomics, epigenomics, and more recently single-cell profiling, provide high-dimensional representations of disease states. These datasets can be complemented by information extracted from public repositories, disease-specific databases, electronic health records, neuroimaging resources, and published literature [4,205,206]. Extensive data reuse, while necessary, introduces important challenges, including batch effects, heterogeneous experimental protocols, incomplete metadata, population biases, missing values, and limited reproducibility across cohorts. Consequently, data harmonization and quality control remain major critical steps in disease model development.

**Figure 3:**
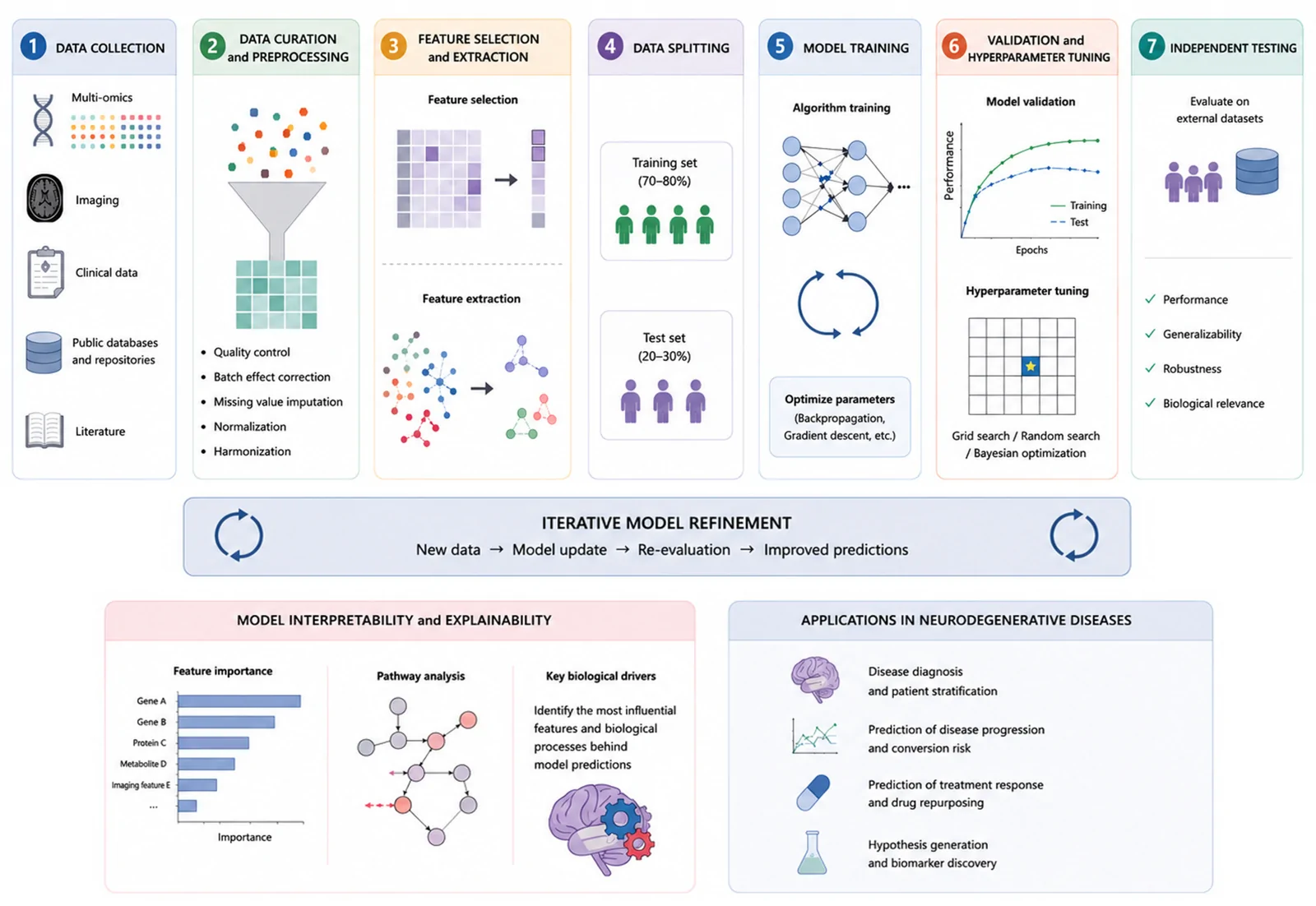
Model construction. Overview of the main steps involved in the development and application of ML models, including (1) data collection from multi-omics, imaging, clinical datasets, public repositories, and literature, (2) data curation and preprocessing, (3) feature selection and extraction, (4) dataset splitting into training and test sets, (5) model training, (6) model validation and hyperparameter tuning, and (7) independent testing on external datasets. The workflow incorporates iterative model refinement through the integration of new data and re-evaluation, as well as model interpretability and explainability approaches to identify key biological drivers underlying model predictions. These computational strategies can support disease diagnosis and patient stratification, prediction of disease progression and conversion risk, treatment-response prediction and drug repurposing, and hypothesis generation/biomarker discovery in NDs.

Model development generally follows a structured workflow (Figure 3) [207,208]. First, large-scale datasets are collected, curated, and preprocessed to minimize technical variability. Features are then selected or extracted through dimensionality reduction or representation learning methods. The resulting datasets are then divided into training, testing, and independent testing cohorts. During training, algorithms iteratively optimize their parameters to capture disease-associated patterns, while validation procedures are used to prevent overfitting and tune model hyperparameters. Finally, model performance is assessed on external datasets to evaluate generalizability and biological relevance. Importantly, explainability methods (i.e., functional analyses) are increasingly incorporated to identify the molecular pathways and biological mechanisms driving model predictions, thereby improving interpretability and facilitating experimental validation.

Classical supervised ML approaches, such as random forests (RF) and support vector machines (SVM), have been widely used to classify patients, identify disease-associated molecular signatures, and predict disease progression. More recently, deep learning architectures, including artificial neural networks (ANNs), autoencoders, graph neural networks (GNNs), and multimodal learning frameworks, have enabled the integration of heterogeneous datasets and the identification of nonlinear relationships that are difficult to capture with conventional statistical methods. In particular, architectures such as convolutional neural networks (CNNs) have been widely applied to neuroimaging, while GNNs have been primarily used to model protein–protein interaction (PPI) networks [209].

In AD, ML models have been developed using combinations of neuroimaging, clinical, genetic, and multi-omics datasets to predict disease onset and cognitive decline trajectories [210]. Deep neural networks trained on multimodal datasets have demonstrated improved performance in capturing disease heterogeneity and generating individualized estimates of disease progression, particularly for patients with mild cognitive impairment at risk of conversion to AD [211,212]. Although these results demonstrate the potential of ML-based approaches for prognostic modeling, their translation into routine clinical practice still requires robust external and prospective validation across heterogeneous patient populations.

Similarly, ML models have been increasingly employed in PD research to integrate molecular biomarkers, neuroimaging, electrophysiological recordings, wearable sensor data, speech analysis and clinical assessments, providing a more comprehensive representation of disease heterogeneity than conventional analytical approaches. These multimodal models have shown promising diagnostic performance, particularly in the early stages of the disease, where subtle pathological changes are often difficult to detect using individual biomarkers alone [213,214]. Beyond diagnosis, ML algorithms have been successfully applied to identify clinically meaningful patient subgroups characterized by distinct molecular and phenotypic profiles, supporting more refined patient stratification and facilitating the implementation of personalized therapeutic strategies. Furthermore, predictive models have been investigated for estimating disease progression, motor and cognitive decline, and the risk of complications such as dyskinesias or cognitive impairment. Recent studies have also highlighted the potential of ML for predicting treatment response, particularly to dopaminergic therapies and DBS, by integrating clinical, imaging, and electrophysiological features [215]. However, many of these applications remain at the research or proof-of-concept stage and require further prospective validation before they can reliably inform individualized therapeutic decisions.

Collectively, these developments show that ML is expanding beyond disease classification toward prognostic modeling, patient stratification, and treatment-response prediction. Some of these applications already provide valuable tools in research and selected clinical settings, whereas their systematic integration into clinical decision-making remains limited. Thus, ML represents a promising component of precision medicine for NDs, provided that model generalizability, interpretability, prospective validation, and clinical utility are rigorously established.

### 4.2. Digital Twins: Toward Predictive and Personalized Models of Neurodegeneration

Emerging computational modeling approaches are moving beyond classification tasks toward the concept of patient-specific digital twins, which can be described as dynamic computational representations of individual patients that integrate longitudinal data and are iteratively updated to simulate disease evolution and system-level responses to pharmacological or environmental perturbations [216]. While this concept is well established in engineering, its implementation in biomedicine, and particularly in NDs, remains at an early stage.

Indeed, the concept of “digital twin” originated in engineering as a virtual representation of a physical asset that is continuously updated using real-time data collected from the corresponding system. In aerospace engineering, for example, a digital twin of an aircraft engine continuously receives information from onboard sensors, allowing the virtual model to reflect the engine’s current state. This continuous synchronization enables engineers not only to monitor system performance but also to simulate future operating conditions, predict component failures, evaluate the impact of maintenance strategies, and optimize system performance before implementing changes in the real physical counterpart. The same concept has been recently translated to biomedicine, where the “physical counterpart” is an individual patient. In this context, a patient’s digital twin could integrate multimodal data acquired throughout disease progression, including genomic, transcriptomic, proteomic, metabolomic, neuroimaging, electrophysiological, and clinical information, as well as data collected from wearable devices and electronic health records. Repeated incorporation of patient-specific information could progressively refine the computational representation of the individual’s biological state. Such models could then be interrogated using in silico simulations to explore clinically relevant scenarios, such as predicting disease progression, estimating the response to surgical/pharmacological interventions, or evaluating alternative therapeutic strategies. In this perspective, digital twins provide a conceptual framework for future patient-specific disease modeling and may eventually contribute to precision medicine and individualized clinical decision-making. As large-scale biomedical repositories continue to grow, digital twins may also benefit from the integration of longitudinal molecular data collected years before the onset of neurodegenerative diseases, even when originally generated for unrelated purposes, providing an unprecedented opportunity to reconstruct disease trajectories from the earliest molecular alterations. However, the extent to which such information can be successfully integrated into clinically actionable patient-specific digital twins remains to be established.

In practice, current biomedical digital twin approaches generally implement only subsets of the features envisioned in the complete digital twin paradigm. They typically combine patient-specific or cohort-derived data with mechanistic or ML-based computational models to reproduce selected aspects of disease progression or treatment response. Thus, the nature of digital twins makes them particularly attractive for NDs, where clinical manifestations and progression are highly heterogeneous and therapeutic responses vary markedly across individuals [217].

Recent applications in AD and PD provide examples of this emerging approach. Bertolini and coworkers [218] developed patient-specific digital twins to model disease progression across the AD spectrum, from mild cognitive impairment to mild-to-moderate AD. Using an unsupervised Conditional Restricted Boltzmann Machine (CRBM) trained on harmonized longitudinal data from cohorts in clinical trials, the authors generated virtual patients sharing the same baseline characteristics as real individuals. The model accurately reproduced the longitudinal evolution of multiple cognitive and functional clinical endpoints simultaneously, despite substantial heterogeneity and missing data. Collectively, these findings demonstrate the feasibility of generating individualized virtual trajectories from longitudinal clinical data and suggest potential applications in the simulation of clinical outcomes and the design of future AD clinical trials. However, this framework does not yet constitute a continuously updated patient-specific digital twin for clinical decision-making and should therefore be regarded primarily as a proof-of-concept implementation.

Another recent example is represented by the work of Angiolelli and coworkers [219], who developed a virtual parkinsonian patient to model the effects of dopaminergic depletion and L-DOPA treatment on whole-brain dynamics. The authors combined a mechanistic whole-brain neural-mass model explicitly integrating DA signaling with EEG and deep brain recordings obtained from patients in both OFF- and ON-medication states and employed Bayesian inference to estimate each individual’s dopaminergic tone. The model was able to reproduce patient-specific changes in large-scale brain dynamics and accurately distinguished the ON and OFF therapeutic conditions. Beyond validating the physiological relevance of the model, this study provides a proof-of-concept demonstration that patient-specific models can reproduce treatment-related changes in brain dynamics. Such approaches could in the future contribute to therapy optimization, including L-DOPA titration or DBS parameter selection.

Despite their promise, several challenges remain before digital twins can become routine research or clinical tools. The construction of accurate patient-specific models requires large, high-quality longitudinal datasets that comprehensively capture disease heterogeneity across multiple biological scales. In addition, as mentioned before, data harmonization, missing information, batch effects, and limited interoperability between public repositories continue to limit model generalizability. Moreover, clinically oriented digital twins will require prospective validation, estimates of uncertainty, interpretable predictions, and evidence that model-informed decisions provide benefits over existing clinical approaches. Future developments will likely rely on increasingly sophisticated multimodal learning strategies coupled with AI, enabling digital twins to generate biologically meaningful hypotheses that can be experimentally verified.

Overall, digital twins represent one of the most ambitious applications of AI in NDs research. Current studies demonstrate the feasibility of constructing patient-specific computational representations of selected aspects of disease progression and therapeutic response, providing important proof-of-concept evidence. The development of fully dynamic digital twins that continuously integrate longitudinal multimodal data and reliably support individualized clinical decisions remains a future objective.

## 5. Conclusions

Neurodegenerative disorders remain among the most challenging diseases to investigate and treat, owing to their remarkable molecular, cellular, and clinical heterogeneity, complex multifactorial etiology, and the limited availability of effective disease-modifying therapies. Consequently, the development of reliable experimental and computational models capable of faithfully reproducing disease mechanisms has become a fundamental prerequisite for understanding pathogenesis, identifying novel therapeutic targets, and accelerating drug discovery.

As discussed throughout this review article, each modeling strategy offers unique strengths while presenting inherent limitations. In vitro systems, particularly patient-derived iPSC-based cultures and organoids, have revolutionized the study of disease mechanisms by enabling the investigation of patient-dependent genetic backgrounds and early pathological processes in a controlled but complex environment. Nevertheless, these models still incompletely recapitulate the cellular diversity, vascularization, immune interactions, and long-term aging processes that characterize the human brain.

In vivo models continue to represent indispensable tools for studying disease progression within the context of a complete organism, allowing investigators to capture complex cell–cell interactions, systemic responses, and behavioral phenotypes that cannot be reproduced in vitro. However, interspecies differences limit the translational value of animal models, contributing to the high failure rate observed, for example, during clinical development of neuroprotective therapies.

Recent advances in computational biology and AI have introduced a complementary paradigm in which in silico models extend beyond descriptive analyses to become predictive frameworks capable of integrating large-scale multimodal datasets, simulating disease progression, and predicting therapeutic responses. Although these approaches remain critically dependent on the quality, diversity, and standardization of the experimental data used for their development, they offer unprecedented opportunities to investigate disease dynamics and support precision medicine through patient-specific predictive models, such as digital twins.

**Figure 4:**
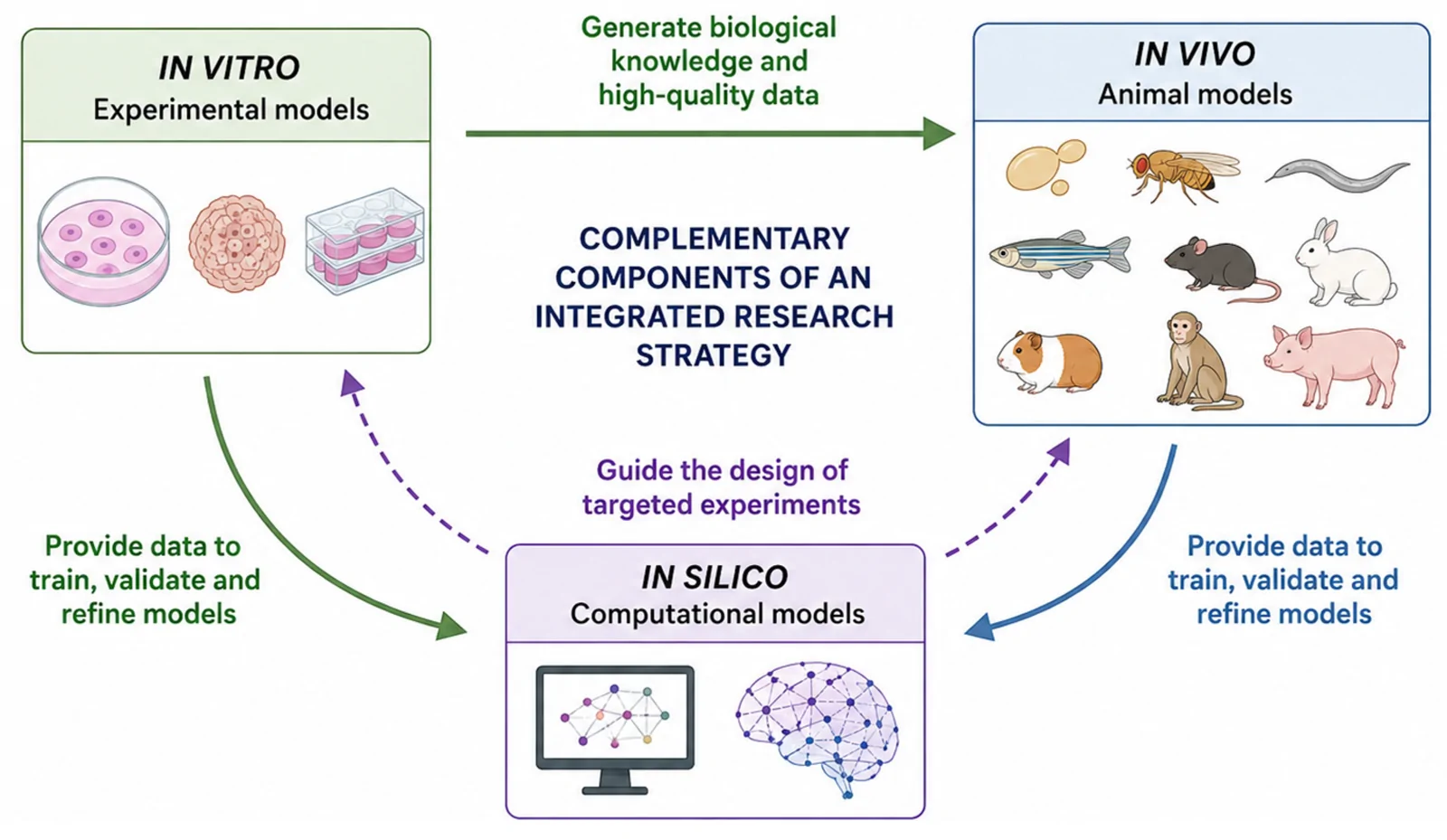
Integration of in vitro, in vivo, and in silico models in NDs research. In vitro and in vivo models generate biological knowledge and high-quality experimental data that can be used to train, validate, and refine computational models. In turn, in silico approaches can guide the design of targeted experiments, establishing an iterative and integrated research strategy. Different modeling paradigms must be considered complementary components of an integrated framework for understanding NDs, improving translational research, and fostering personalized medicine.

Rather than representing competing methodologies, in vitro, in vivo, and in silico models should be viewed as complementary components of an integrated research strategy (Figure 4). Experimental models generate the biological knowledge and high-quality datasets required to train, validate, and continuously refine computational models, while in silico predictions can guide the design of increasingly targeted experimental studies, reducing costs, shortening development timelines, and minimizing animal use.

As multimodal data generation and ML methodologies continue to evolve, the integration of experimental and computational approaches may provide an increasingly valuable framework for future ND research, with the potential to improve disease modeling, support patient stratification, and ultimately contribute to the development of more personalized and effective disease-modifying interventions.

Importantly, this integrative framework should not be confined to the integration among in vitro, in vivo, and in silico models, but should increasingly incorporate clinical observations and the parallel evolution of pharmacological and surgical interventions. Clinical data can inform and refine disease models, while insights generated by experimental and computational approaches can, in turn, support patient stratification, therapeutic development, and treatment optimization. In this context, a particularly promising direction for NDs research lies in strengthening the bidirectional connection between computational modeling and bedside clinical practice, ultimately facilitating the translation of mechanistic knowledge into more individualized therapeutic strategies.

## Figures and Tables

**Table 1:** List of immortalized/tumor-derived human cell lines for NDs modeling. Overview of the disease relevance, the key strengths (✓), limitations (×), and primary applications (→).

Cell Line	Disease(s) Relevance	Strengths (✓) & Limitations (✗)	Applications (→)	Key References
SH-SY5Y Human neuroblastoma	AD, PD, ALS, FTD	✓ Can be differentiated into catecholaminergic/dopaminergic phenotype; mimick DA dyshomeostasis upon DA supplementation ✗ Tumor origin; immature neuronal phenotype	→ exogenous DA toxicity and dyshomeostasis studies; mitochondrial dysfunction assays; protein aggregation assays; cytotoxicity and BBB permeability assays	[13,18,19,20,21]
LUHMES Human fetal ventral mesencephalon-derived neural progenitors	PD	✓ High reproducibility; robust dopaminergic phenotype ✗ Limited use for non-dopaminergic diseases	→ gold standard for PD modeling; protein aggregation assays; mitochondrial dysfunction assays	[22,23,24]
HEK293/HEK293T Human embryonic kidney	AD, PD, ALS, HD	✓ High transfection efficiency; ease of genetic manipulation; ✗ Non-neuronal phenotype; poor physiological relevance to neurodegeneration	→ high-yield recombinant protein expression; packaging platform for AAV and lentiviral vectors (HEK293T)	[25,26,27,28]
SK-N-BE(2)/BE(2)-M17 Human neuroblastoma	AD, PD	✓ Can be differentiated into catecholaminergic/dopaminergic phenotype ✗ Tumor origin; immature neuronal phenotype	→ endogenous catecholaminergic modeling; protein aggregation assays	[29,30]
HNSC.100 Human neural stem cell	PD	✓ Multipotent ✗ Poorly characterized; limited use	→ multilineage ND modeling (neurons, astrocytes, and oligodendrocytes); transcriptomic profiling of expressed disease-related genes; genetic manipulation and disease-gene screening	[31]
ReNcell VM/CX Human ventral mesencephalon neural progenitor cells	AD, PD, ALS, HD	✓ Differentiates into neurons and astrocytes; midbrain identity ✗ Heterogeneous differentiation; expensive	→ mitochondrial dysfunction assays; protein aggregation assays; compatible with three-dimensional (3D) extracellular matrices	[9,32,33]
HB1.F3 Human fetal telencephalon neural stem cell	AD, PD, ALS, HD	✓ Multipotent ✗ Limited physiological relevance	→ multilineage ND modeling (neurons, astrocytes, and oligodendrocytes); multiple engineered variants; suitable for transplantation and neuroprotection assays	[34,35,36]
CTX0E03 Human fetal cortical neural stem cells	AD, HD	✓ Multipotent; GMP-compliant ✗ Primarily used for cell-therapy rather than model ND cellular pathology	→ clinical-grade stem cell therapy and tissue repair models; paracrine neuroprotection and host tissue regeneration assays; mechanistic studies on lesion recovery	[37]
U87-MG Human glioblastoma	AD, HD	✓ Glial/astrocytic origin ✗ Non-neuronal; tumor origin	→ non-cell-autonomous disease spreading and intercellular transport models; astrocyte-like clearance and neuroinflammatory response assays	[38,39]
HMC3 Human embryonic microglia	AD, PD	✓ Standardized human microglial model with robust expression of microglial homeostatic and activation markers ✗ Non-neuronal	→ human-specific microglial neuroinflammatory response models; pathological protein-induced senescence and inflammation; anti-inflammatory and neuroprotective compounds screening	[40,41]
MO3.13 Human oligodendrocyte–rhabdomyosarcoma hybrid cell line	AD, PD	✓ Oligodendroglial model ✗ Non-neuronal, hybrid/tumor origin; cannot achieve structural myelination in 2D monoculture without neuronal axonal co-culture	→ oligodendrocyte pathology, lipid metabolism, and remyelination screening in NDs; non-cell-autonomous myelin protection and neurotrophic signaling mechanisms; pro-myelinating and oligodendrocyte-protective drugs screening	[42,43]

**Table 2:** Comparative summary of in vitro models for NDs research. Overview of the key strengths (✓), limitations (×), and primary applications (→) of each model.

In Vitro Model	Strengths (✓) & Limitations (✗)	Applications (→)
Tumor-Derived & Immortalized Cell Lines	✓ Commercially available, low-maintenance, and highly cost-effective; high reproducibility. ✗ Genomic instability, reduced physiological/metabolic fidelity, and limited availability for rare NDs subtypes.	→ Baseline mechanistic studies, high-throughput compound screening, and acute exogenous stress modeling (e.g., oxidative stress, toxicity insults).
Primary Rodent Neuronal Cultures	✓ Genetically stable; mature OXPHOS metabolism, functional synapses, and gold-standard dendritic spine architecture. ✗ Translational gap: significant interspecies variability limits; high batch variability (embryonic age/isolation), short lifespan (2–4 weeks), and lack of cellular aging hallmarks.	→ Acute synaptic toxicity assays, electrophysiological validations, and functional ion channel/synapse mechanics.
Human-Induced Pluripotent Stem Cells (hiPSC)- Derived Neurons	✓ Patient-specific genetic fidelity, bypasses tissue/ethical barriers and facilitates autologous modeling and pre-symptomatic stratification. ✗ Long-term genomic instability, high donor polymorphism, and loss of age-associated epigenetic signatures due to reprogramming.	→ Patient-specific target discovery, gene mutation validation, and personalized drug evaluation.
3D Organoids	✓ Recapitulates 3D cytoarchitecture, enables region-specific selective vulnerability and cell interactions, and integrates with single-cell multi-omics. ✗ Expensive, long culture durations, lack of functional vascularization (necrotic core), incomplete adult maturation, and limited protocol standardization.	→ Localized cell-type vulnerability, non-cell-autonomous glial-neuronal pathogenesis, and tissue-level protein aggregation.
3D Assembloids	✓ Models long-range connectivity and inter-regional crosstalk, enabling advanced functional testing and single-cell multi-omics compatibility ✗ Share organoid constraints plus complex assembly and standardization challenges.	→ Long-range circuit dynamics, inter-regional aggregate propagation, and blood–brain barrier (BBB) functional modeling.
*Post-Mortem* Brain Tissues & Organotypic Slice Cultures (OSCs)	✓ Gold-standard biological reference offering fully mature human cells, end-stage histopathology, and non-cell-autonomous pathology context (post-mortem brain tissue); retains authentic 3D cytoarchitecture, cellular heterogeneity, and mature synaptic networks for high-fidelity ex vivo translational benchmarking (OSCs). ✗ Static endpoint snapshot lacking functional assays, restricted sample availability, high donor variability, and post-mortem delay artifacts (post-mortem brain tissue); mechanical tissue damage, limited long-term viability, high logistical hurdles, and low throughput (OSCs).	→ End-stage target validation, histopathologic benchmarking, and molecular verification of in vitro model findings (post-mortem brain tissue). → Longitudinal aggregate propagation tracking, acute circuit-level functional assays, and short-term toxicological benchmarking (OSCs).
Patient-Derived Biofluids	✓ Minimally invasive and highly accessible platform. ✗ Function purely as diagnostic/prognostic tool; cannot physically model cellular or tissue disease mechanisms.	→ Biomarker discovery, pre-symptomatic diagnosis, patient stratification, and longitudinal disease tracking.

**Table 3:** List of in vivo models for NDs. Overview of the key strengths (✓), limitations (×), and primary applications (→) of each model.

Model	Strengths (✓) & Limitations (✗)	Applications (→)	Key References
Yeast (*S. cerevisiae*)	✓ Low-cost, easy genetic manipulation via homologous recombination; strong conservation of eukaryotic pathways. ✗ Lack of multicellularity/tissue context; absence nervous system; no behavioral or disease-progression readouts; limited translatability.	→ Protein misfolding/aggregation studies. → Mitochondrial proteostasis dysfunction and Aβ processing. → High-throughput drug/genetic screening.	[116,117,118,119,120,121,122,123]
Non-mammalian models (*D. melanogaster*, *C. elegans*, *D. rerio*)	✓ Low-cost, easy husbandry, rapid life cycle; high genetic orthology with humans. ✗ Simple nervous system and limited neurological readouts; anatomical divergence; limited direct translational potential.	→ Real-time in vivo visualization of protein aggregation and neuronal loss. → Locomotor/motor phenotype screening. → Neuroinflammation and innate immune activation studies. → Dopaminergic neurodegeneration modelling (MPTP-zebrafish; SNCA/LRRK2-C. elegans). → Gut-brain axis and microbiome-neurodegeneration studies.	[124,125,126]
Small mammalian models (mouse, rat, rabbit, guinea pig)	✓ Conserved CNS organization; easy genetic manipulation; validated behavioral/cognitive testing tools; comparatively longer lifespan supports progressive disease modelling; can host humanized systems (e.g., rat-organoid chimeras). ✗ Lower throughput/higher cost than non-mammals; short lifespan limits full age-related pathology; genomic/physiological differences from humans; lack of standardized tools for rabbits/guinea pigs; incomplete pathological phenotypes.	→ Protein aggregation and prion-like spreading. → Neuroinflammation and immune–nervous system crosstalk. → Cognitive and behavioral decline modelling. → Preclinical drug efficacy and safety screening. → Source of primary neuronal/glial cultures for phenotypic assays.	[127,128,129,130,131,132,133,134]
Large mammalian models (Non-human primates, pigs)	✓ High anatomical/structural similarity to human brain connectivity; long lifespan; complex cognitive/behavioral repertoire. ✗ Significant ethical/regulatory constraints; high maintenance costs; small sample sizes limit statistical power; technical challenges in genome editing; high phenotypic variability; low throughput due to long timelines.	→ Spontaneous age-related neuropathology, modeling sporadic NDs. → Overt, selective neurodegeneration (nigrostriatal, striatal medium spiny neuron loss). → Clinically translatable neuroimaging (MRI/PET), DBS and optogenetic studies. → Preclinical gene/cell therapy biodistribution and immunogenicity testing. → Gut-brain axis studies.	[135,136,137,138,139,140,141,142]

## Data Availability

No new data were created or analyzed in this study. Data sharing is not applicable to this article.

## Abbreviations

The following abbreviations are used in this manuscript:

3R τ	Three-repeat tau isoform
4R τ	Four-repeat tau isoform
AD	Alzheimer’s disease
AI	Artificial intelligence
ALS	Amyotrophic lateral sclerosis
ANN	Artificial neural network
APP	Amyloid precursor protein
Aβ	Amyloid-beta
Aβ40	Amyloid-beta 40
Aβ42	Amyloid-beta 42
BBB	Blood–brain barrier
BDNF	Brain-derived neurotrophic factor
CNN	Convolutional neural network
CNS	Central nervous system
CO	Cortical organoid
CRBM	Conditional Restricted Boltzmann Machine
CSF	Cerebrospinal fluid
DA	Dopamine/Dopaminergic
DBS	Deep brain stimulation
DPR	Dipeptide repeat
EBiSC	European Bank for induced Pluripotent Stem Cells
EEG	Electroencephalography
FTD	Frontotemporal dementia
GA	Glycine–alanine (poly(GA) dipeptide repeat)
GBA	Gut–brain axis
GBA1	Glucocerebrosidase beta 1
GMP	Good Manufacturing Practice
GNN	Graph neural network
HD	Huntington’s disease
hiPSCs	Human-induced pluripotent stem cells
HMC3	Human microglial cell line HMC3
HTT	Huntingtin
IL6	Interleukin-6
L-DOPA	Levodopa (L-3,4-dihydroxyphenylalanine)
LPS	Lipopolysaccharide
LRRK2	Leucine-rich repeat kinase 2
LUHMES	Lund human mesencephalic cells
MISCO	Midbrain–Striatal–Cortical Organoid
ML	Machine learning
MO	Midbrain organoid
MPTP	1-Methyl-4-phenyl-1,2,3,6-tetrahydropyridine
MRI	Magnetic resonance imaging
MS	Multiple sclerosis
MSN	Medium spiny neuron
ND	Neurodegenerative disease
NHP	Non-human primate
OFF	Off medication state
ON	On medication state
OSC	Organotypic slice culture
OXPHOS	Oxidative phosphorylation
PBMC	Peripheral blood mononuclear cell
PD	Parkinson’s disease
PET	Positron emission tomography
PFF	Preformed fibril
PPI	Protein–protein interaction
RF	Random forest
SCNT	Somatic cell nuclear transfer
scRNA-seq	Single-cell RNA sequencing
snRNA-seq	Single-nucleus RNA sequencing
SVM	Support vector machine
XCI	X-chromosome inactivation
α-syn	Alpha-synuclein
β-amyloid	Beta-amyloid
τ	Tau protein
